# Titrimetric and Spectrophotometric Methods for the Assay of Ketotifen Using Cerium(IV) and Two Reagents

**DOI:** 10.1155/2013/697651

**Published:** 2013-11-12

**Authors:** Madihalli Srinivas Raghu, Kanakapura Basavaiah, Kudige Nagaraj Prashanth, Kanakapura Basavaiah Vinay

**Affiliations:** Department of Chemistry, University of Mysore, Manasagangotri, Mysore, Karnataka 570006, India

## Abstract

One titrimetric and two spectrophotometric methods are described for the determination of ketotifen fumarate (KTF) in bulk drug and in tablets using cerium(IV) as the oxidimetric agent. In titrimetry (method A), the drug was treated with a measured excess of cerium(IV) in H_2_SO_4_ medium and after a standing time of 10 min, the surplus oxidant was determined by back titration with iron(II). The spectrophotometric procedures involve addition of a known excess of cerium(IV) to KTF in acid medium followed by the determination of unreacted oxidant by reacting with either *p*-dimethyl amino benzaldehyde and measuring the resulting colour at 460 nm (method B) or *o*-dianisidine and subsequent measurement of the absorbance of coloured product at 470 nm (method C). Titrimetric assay is based on a 1 : 2 reaction stoichiometry between KTF and cerium(IV) and the method is applicable over 2–18 mg range. In spectrophotometry, regression analysis of Beer's law plots showed a good correlation in 0.4–8.0 and 0.4–10.0 g mL^−1^ KTF ranges for method B and method C, respectively, and the corresponding molar absorptivity coefficients are calculated to be 4.0 × 10^4^ and 3.7 × 10^4^ L mol^−1^ cm^−1^.

## 1. Introduction

**Figure 1:**
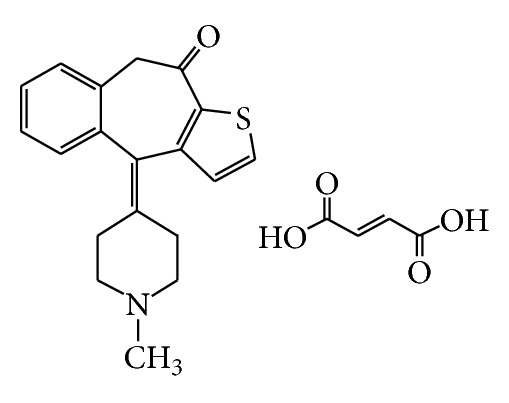
Structure of KTF.

Ketotifen fumarate (KTF) (Figure 1), chemically known as 4,9-dihydro-4-(1-methyl-4-piperidinylidene)-10H-benzo[4,5]cyclohepta-[1,2-b] thiophen-10-one, which belongs to the group of cyclohepta thiophenones is an antiallergic drug with stabilizing action on mast cells, analogous to that of sodium cromoglycate, and anti-H_1_ effect [1]. Ketotifen is given orally as fumarate in the prophylactic management of asthma and is used in the treatment of allergic conditions such as rhinitis and conjunctivitis [2]. Ketotifen is a nonbronchodilator, antiallergic properties, and has a specific anti-H_1_ effect [3]. 

The drug is official in British Pharmacopoeia [4], which describes a potentiometric titration with perchloric acid in acetic anhydride-acetic acid medium. The therapeutic importance of the drug has prompted the development of a variety of techniques for its assay in pharmaceuticals and body fluids. In pharmaceuticals, it is quantified by high performance liquid chromatography [4–10], high performance thin layer chromatography [11], liquid chromatography with tandem mass spectrometry [12], capillary electrophoresis [13], spectrofluorimetry [14], direct differential pulse polarographic and adsorptive-stripping voltammetry [15], and UV spectrophotometry [16, 17]. Potentiometric titration methods employing polymeric membrane sensors were also developed, where the method involved potentiometric titration of the drug with sodium tetraphenylborate [18, 19]. Determination of KTF has also been carried out based on chemiluminescence [20–22]. Coulometric titration of KTF in pure drug and in tablet forms using biamperometric end-point detection was developed by Ciesilski et al. [23].

To the best of our knowledge, no visual titrimetric method has ever been reported for KTF. Visual titrimetry and visible spectrophotometry may serve as a useful alternatives to many of the aforesaid sophisticated techniques because of their cost-effectiveness, ease of operation, sensitivity, fair accuracy, precision, and wide applicability.

 Several visible spectrophotometric methods based on colour reactions involving the amino or thiophene of the ketotifen molecule can be found in the literature. El-Kousy and Bebawy [24] have developed three methods for the assay of KTF in bulk drug and in tablets; the first method was based on extraction of drug-cobalt thiocyanate ternary complex with methylene chloride and estimation by indirect atomic absorption method via the determination of the cobalt content in the complex after extraction in 0.1 M hydrochloric acid at 240.7 nm. The second method is based on the formation of orange red ion pair by the reaction of KTF and molybdenum thiocyanate with absorption maxima at 469.5 nm in dichloromethane. The third method was a charge transfer reaction between drug and 2,3-dichloro-5,6-dicyano-p-benzoquinone in acetonitrile medium. Sastry and Naidu [25] have determined the drug in tablets and syrup based on the reduction of Folin-Ciocalteau reagent to a blue coloured chromogen measurable at 720 nm, in the same article; KTF was treated with a measured excess of N-bromosuccinimide (NBS), the unreacted oxidant was reacted with celestine blue, and the change in absorbance was measured at 540 nm. The third method was based on the formation of colored species by the coupling of the diazotized sulphanilamide, and absorbance of the colored species was measured at 520 nm.

 There are three reports on the use of ion-pair reaction for the assay of KTF. An extractive spectrophotometric procedure was based ion-pair reaction using Azocarmine G as ion-pair reagent at pH 1.5. The ion-pair complex extracted into CHCl_3_ was measured at 540 nm [25]. Sane et al. [26] have described four extractive spectrophotometric procedures based on this reaction using bromophenol blue, bromothymol green, bromothymol blue and bromocresol purple, as ion-pair reagents in acidic medium. The drug has also been determined spectrophotometrically based on ion-pair complex formation with bromocresol green [27] at pH 3.0 followed by extraction into chloroform and measurement at 423 nm. Beer's law is obeyed over the concentration range 5.15–61.91 *μ*g mL^−1^ KTF. The charge-transfer reaction of KTF with picric acid was developed by Vachek [28]. The method involves extraction of the picric acid-drug complex into chloroform and measurement of the extract at 405 nm. 

The reported spectrophotometric methods suffer from some other disadvantage such as narrow linear range, poor sensitivity, dependence on critical experimental variables, tedious and time-consuming extraction steps, heating step, and/or use of expensive reagent or large amounts of organic solvents as indicated in Table 1.

 From the foregoing paragraphs, it is clear that cerium(IV) despite its strong oxidizing power, versatility, and high stability in solution has not been applied for the assay of ketotifen. This paper describes for the first time the application of acidic cerium(IV) to the titrimetric and spectrophotometric determination of KTF using *p*-dimethyl amino benzaldehyde and *o*-dianisidine as chromogenic agents. cerium(IV) has earlier been widely applied for the assay of a variety of pharmaceuticals [29–33].

## 2. Experimental

### 2.1. Apparatus

A Systronics model 106 digital spectrophotometer (Systronics, Ahmedabad, Gujarat, India) with 1 cm path length matched quartz cells was used to record the absorbance values.

### 2.2. Materials

All chemicals used were of analytical reagent grade. Double distilled water was used throughout the investigation. Pharmaceutical grade KTF (99.78 percent pure) was procured from Cipla India, Ltd., Mumbai, India, as a gift and used as received. Asthafen-1 (Torrent pharmaceuticals, Sikkim, India) and Ketasma-1 (Sun pharmaceuticals, Sikkim, India) tablets were purchased from local commercial market.

### 2.3. Reagents and Chemicals

Cerium(IV)* Sulphate (0.005 M)*. An approximately 0.01 M solution was prepared by dissolving about 2.52 g of the chemical Ce(SO_4_)_2_·4H_2_O (Loba Chemie Pvt. Ltd., Mumbai, India) in 0.5 M sulphuric acid with the aid of heat and diluting to 250 mL with the same acid and standardized using standard ferrous ammonium sulphate solution (FAS) [34]. It was used for titrimetric work and diluted to obtain working concentrations of 300 and 100 *μ*g mL^−1^ cerium(IV) for spectrophotometric methods B and C, respectively, with the same solvent.

*Perchloric Acid (4 M)*. Perchloric acid (4 M) was prepared by diluting appropriate volume of commercially available acid (70%; Merck, Mumbai, India) with water.

*Sulphuric Acid (5 M)*. A 5 M sulphuric acid was prepared by appropriate dilution of concentrated acid (98%; Sp. gr. 1.84, Merck, Mumbai, India) with water and used in both methods B and C.

*Ferroin Indicator*. It was prepared by dissolving 0.742 g of 1,10-phenanthroline monohydrate in 50 mL of 0.025 M ferrous sulphate solution (0.348 g of ferrous sulphate heptahydrate in 50 mL water).

*p-Dimethylamino Benzaldehyde (p-DMAB) (0.5%)*. An accurately weighed 0.5 g of *p*-DMAB (Merck, Mumbai, India) was transferred to a 100 mL volumetric flask and dissolved in 4 M HClO_4_ and the volume was made up to the mark with the same solvent.

*o-Dianisidine (ODS) (0.05%)*. An accurately weighed 50 mg of *o*-dianisidine (Merck, Mumbai, India) was transferred to a 100 mL volumetric flask and dissolved in ethanol and the volume was made up to the mark with the same solvent.

*Standard KTF Solution*. A stock standard solution equivalent to 2.0 mg mL^−1^ KTF was prepared by dissolving 200 mg of pure drug with water in a 100 mL calibrated flask. This solution was used in the titrimetric work and a working concentration of 20 *μ*g mL^−1^ was prepared by stepwise dilution with the same acid and used in method C. For method B, 200 *μ*g mL^−1^ KTF solution was prepared separately by dissolving accurately weighed 20 mg of pure drug in 4 M HClO_4_ and diluting to volume with the same solvent in a 100 mL standard flask, and the resulting solution (200 *μ*g mL^−1^) was diluted to 20 *μ*g mL^−1^ with 4 M HClO_4_ and used for assay.

## 3. Assay Procedures

### 3.1. Titrimetry (Method A)

A 10 mL aliquot of the KTF solution containing 2–18 mg of KTF was transferred into a 100 mL conical flask. To this, 10 mL of 0.005 M cerium(IV) sulphate was added using a pipette, and the contents were mixed well and the flask set aside for 10 min. Finally, the unreacted oxidant was titrated with 0.005 M FAS solution using one drop of ferroin indicator. Simultaneously, a blank titration was performed, and the amount of the drug in the measured aliquot was calculated from the amount of cerium(IV) reacted.

The amount of KTF in the aliquot was calculated using the formula

(1) $$\begin{array}{c} \text{Amount}\;\;\left(\text{mg}\right)=\frac{\left\{\left(B-S\right)\times M_{w}\times C\right\}}{n}, \end{array}$$

where *B* = volume of FAS consumed in the blank titration, mL. *S* = volume of FAS consumed in the sample titration, mL. *M*_*w*_= relative molecular mass of KTF. *C* = molar concentration or strength of oxidant in mol L^−1^. *n* = number of moles of Ce(IV) reacting with each mole of KTF.

### 3.2. Spectrophotometry Using *p*-Dimethylaminobenzaldehyde (Method B)

Different aliquots (0.2,0.5,…, 4 mL) of standard 20 *μ*g mL^−1^ KTF solution in 4 M HClO_4_ were transferred into a series of 10 mL calibrated flasks by means of a microburette and the total volume in all the flasks was adjusted to 4 mL by adding 4 M HClO_4_. To each flask, 2 mL of 5 M H_2_SO_4_ and 1 mL of 300 *μ*g mL^−1^ Ce(IV) solution were added, and the content was mixed well. After a standing time of 10 min, 1 mL of 0.5% *p*-DMAB was added to each flask and the volume was made up to mark with 4 M HClO_4_. After 5 min, the absorbance of the each coloured product was measured at 460 nm against water.

### 3.3. Spectrophotometry Using *o*-Dianisidine (Method C)

Varying aliquots (0.20,0.5,…, 5.0 mL) of a standard 20 *μ*g mL^−1^ KTF solution were transferred into a series of 10 mL volumetric flasks using a microburette and the total volume was brought to 5 mL by adding water. To each flask, 1 mL of 5 M H_2_SO_4_ followed by 1 mL of 100 *μ*g mL^−1^ Ce(IV) solution were added; and the content was mixed well and kept aside for 10 min at ambient temperature. Finally, 1 mL of 0.05% *o*-dianisidine was added to each flask, the volume was made up to mark with water and mixed well. The absorbance of each solution was measured at 470 nm against water blank after 5 min.

A standard graph was prepared by plotting absorbance against concentration, and the unknown concentration was read from the graph or computed from the regression equation derived using Beer's law data.

### 3.4. Procedure for the Analysis of Tablet

Two hundred tablets were weighed and finely powdered. An accurately weighed quantity of the tablet powder equivalent to 100 mg KTF was transferred into a 50 mL calibrated flask and about 30 mL water. The content of the flask was shaken for 20 min, and finally the volume was completed to the mark with water. The content was mixed well and filtered through a Whatman No. 42 filter paper. First, 5 mL portion of the filtrate was discarded, and a suitable aliquot of the filtrate (2 mg mL^−1^ KTF) was then subjected to titrimetric analysis (method A). This tablet extract was diluted stepwise to get 20 *μ*g mL^−1^ KTF with water, used in spectrophotometry (method C), by following the procedure described earlier.

Tablet extract equivalent to 20 *μ*g mL^−1^ KTF was prepared separately using 4 M HClO_4_ and subjected to analysis in spectrophotometric method B by following the assay procedure described earlier. 

### 3.5. Procedure for the Analysis of Placebo Blank and Synthetic Mixture

 A placebo blank containing starch (30 mg), acacia (35 mg), hydroxyl cellulose (35 mg), sodium citrate (40 mg), talc (30 mg), magnesium stearate (45 mg), and sodium alginate (35 mg) was prepared by combining all components to form a homogeneous mixture. A 50 mg of the placebo blank was accurately weighed and its solution was prepared as described under “tablets” and then subjected to analysis by following the general procedure.

 A synthetic mixture was prepared by adding an accurately weighed 100 mg of KTF to about 150 mg of the placebo mentioned above. The extraction procedure applied for tablets was applied to prepare 2 mg mL^−1^ KTF solution. This was diluted to get 20 *μ*g mL^−1^ KTF solutions with respective solvents and used in spectrophotometric methods. Three different volumes of the resulting synthetic mixture solution were subjected to analysis by following the respective general procedure.

## 4. Results and Discussion

KTF is reported to undergo oxidation with NBS [22], and also cerium(IV) has been used as an oxidimetric reagent for the assay of many oxidisable drugs [29–33]. The present work is based on the oxidation of the sulphur atom of the KTF molecule by a measured excess of cerium(IV) in acid medium and the surplus oxidant was determined by either titrimetry or spectrophotometry.

### 4.1. Titrimetry

KTF was found to react with Ce(IV) sulphate in sulphuric acid medium. The titrimetric method (method A) involves oxidation of KTF by a known excess of Ce(IV) in sulphuric acid medium with the formation of ketotifen sulphoxide and the unreacted oxidant was determined by back titration with FAS. H_2_SO_4_ medium was favored over HCl and HClO_4_ medium since the reaction was found to yield a regular stoichiometry in the concentration range studied. Reproducible and regular stoichiometry was obtained when 0.65–1.29 M H_2_SO_4_ concentration was maintained. Hence, 5 mL of 5 M H_2_SO_4_ solution in a total volume of 25 mL (1 M H_2_SO_4_ overall) was found to be the most suitable concentration for the quantitative reaction between KTF and Ce(IV). Under the optimized reaction condition, there was found to be a definite reaction stoichiometry of 1 : 2 between KTF and Ce(IV) within the range of 2–18 mg of KTF. 

### 4.2. Spectrophotometry

In spectrophotometry (method B), the unreacted Ce(IV) was treated with *p*-DMAB in HClO_4_ medium to yield formic acid and *p*-dimethylaminophenol, which upon further oxidation gave the corresponding quinoimine derivative [35], measured at 460 nm, and in method C, the surplus oxidant was treated with *o*-dianisidine in acid medium to form the orange red colour product and measured at 470 nm.

In methods B and C, the increasing concentrations of drug added to a fixed concentration of Ce(IV) yield decreasing concentrations of Ce(IV), and this concomitant decrease in concentration results in decreasing absorbance values of its reaction product with *p*-dimethylaminobenzaldehyde in method B and with *o*-dianisidine in method C.

## 5. Method Development

### 5.1. Absorption Spectra

**Figure 2:**
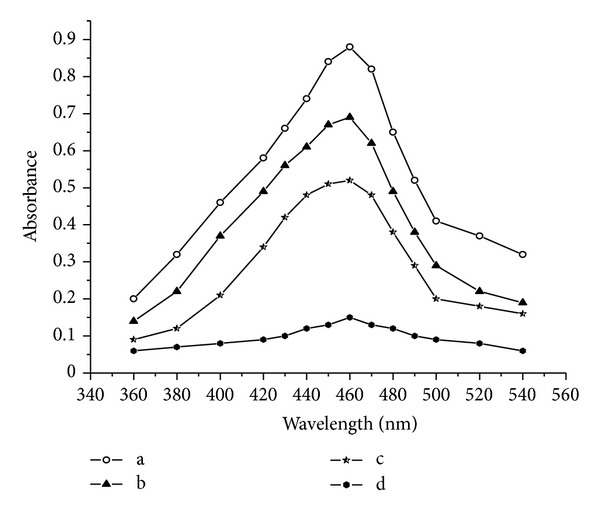
Absorption spectra, method B, (a, b, c, and d are absorption spectra of blank, 2, 4, and 8, 20 *μ*g mL^−1^ KTF, resp.).

The addition of *p*-DMAB to Ce(IV) resulted in the formation of yellowish red coloured product with maximum absorption at 460 nm against blank, and the decrease in the absorption intensity at 460 nm, caused by the presence of the drug, was directly proportional to the amount of the drug reacted in method B shown in Figure 2.

**Figure 3:**
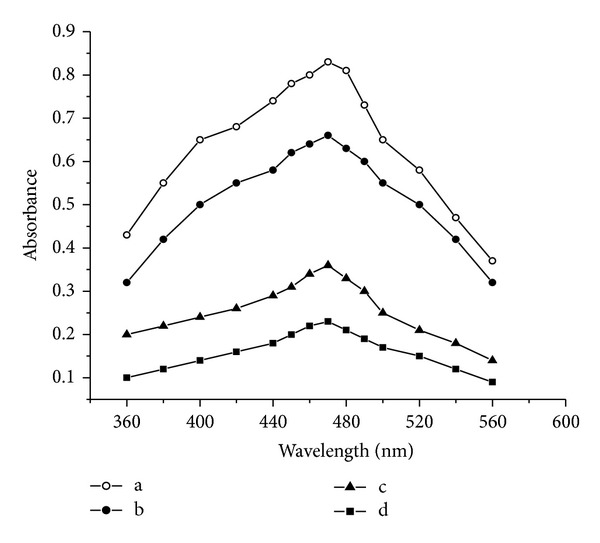
Absorption spectra, method C, (a, b, c, and d are absorption spectra of, blank, 2, 6, and 8 *μ*g mL^−1^ KTF, resp.).

In method C, the orange coloured product of Ce(IV) with *o*-dianisidine shows maximum absorption at 470 nm against its corresponding reagent blank as shown in Figure 3, and the decrease in the absorption intensity at 470 nm, caused by the presence of the drug, was directly proportional to the amount of the drug.

### 5.2. Selection of Reaction Medium

Perchloric acid (4 M) medium was found ideal for rapid and quantitative reaction between KTF and Ce(IV) and to obtain maximum and constant absorbance values due to Ce(IV)-*p*-DMAB reaction product at 460 nm. This may be attributed to the highest oxidation potential of Ce(IV) in HClO_4_ (*E*_*o*_ = 1.75 V) as compared to that of Ce(IV) in H_2_SO_4_ (*E*_*o*_ = 1.44 V), HNO_3_ (*E*_*o*_ = 1.61 V), or HCl (*E*_*o*_ = 1.28 V) [36]. Therefore, all the solutions (ALB, Ce(IV), and *p*-DMAB) were prepared in 4 M perchloric acid throughout the investigation and the same was maintained as reaction medium in method B.

### 5.3. Optimization of Ce(IV) Concentration for Methods B and C

In method B, to fix the optimum concentration of Ce(IV), different concentrations of oxidant were reacted with a fixed concentration of *p-*DMAB in HClO_4_ medium and the absorbance was measured at 460 nm. A constant and maximum absorbance resulted was 30 *μ*g mL^−1^ Ce(IV), and hence different concentrations of KTF were reacted with 1 mL of 300 *μ*g mL^−1^ Ce(IV) in HClO_4_ medium before determining the residual Ce(IV). This facilitated the optimization of the linear dynamic range over which procedure could be applied for the assay of KTF. In method C, 10 *μ*g mL^−1^ Ce(IV) gave a constant and maximum absorbance with* o*-dianisidine at 470 nm, and hence 1 mL of 100 *μ*g mL^−1^ Ce(IV) was fixed as optimum. 

### 5.4. Study of Reaction Time and Stability of the Coloured Species

Under the described experimental conditions, the reaction between KTF and Ce(IV) was complete within 10 min at room temperature (28 ± 2°C). After the addition of *p-*DMAB, a standing time of 5 min was necessary for the formation of coloured product, and thereafter the absorbance of the coloured product was stable for more than an hour in method B.

 In method C, with fixed concentrations of KTF, Ce(IV), ODS, and acid, the absorbance was measured by different intervals of time which showed that reaction time is five minutes, and the coloured product is stable for 15 min.

### 5.5. Effect of Diluent

In order to select proper solvent for dilution, different solvents were tried. The highest absorbance values were obtained when 4 M HClO_4_ was used as diluting solvent, and substitution of 4 M HClO_4_ by other solvents (methanol, water, 6 M HClO_4_) resulted in decrease in the absorbance values in method B. In method C, *o*-dianisidine is prepared in ethanol and drug in water, and water as a diluent gave maximum absorbance.

## 6. Method Validation

### 6.1. Linearity and Sensitivity

 The present methods were validated for linearity, selectivity, precision, accuracy, robustness, ruggedness, and recovery. Over the range investigated (2–18 mg), a fixed stoichiometry of 1 : 1 [KTF : Ce(IV)] was obtained in titrimetry which served as the basis for calculations. In spectrophotometry, the calibration graphs were found to be linear from 0.4–8.0 *μ*g mL^−1^ and 0.4–10.0 *μ*g mL^−1^ KTF in method B and method C, respectively, in the inverse manner.

 The calibration graphs are given by the equation

(2) $$\begin{array}{c} Y=a+bX, \end{array}$$

(where, *Y* = absorbance, *a* = intercept, *b* = slope, and *X* = concentration in *μ*g mL^−1^) obtained by the method of least squares. Sensitivity parameters such as molar absorptivity and Sandell's sensitivity values and the limits of detection (LOD) and quantification (LOQ) were calculated according to the ICH guidelines [37] using the following formulae:

(3) $$\begin{array}{c} \text{LOD}=3.3\frac{S}{\text{slope}},\;\;\text{LOQ}=10\frac{S}{\text{slope}}, \end{array}$$

where *S* is the standard deviation of the absorbance of seven blank readings. These are summarized in Table 2.

### 6.2. Accuracy and Precision

 The repeatability of the proposed methods was determined by performing five replicate determinations. The intra-day and inter-day variation in the analysis of KTF was measured at three different levels. The accuracy of an analytical method expresses the closeness between the reference value and the found value. Accuracy was evaluated as percentage relative error between the measured and taken amounts/concentrations. The results of this study are compiled in Table 3 and speak of the excellent intermediate precision (%RSD ≤ 2.90) and accuracy (%RE ≤ 2.42) of the results.

### 6.3. Robustness and Ruggedness

 To evaluate the robustness of the methods, two important experimental variables, namely, standing time and volume of H_2_SO_4_, were slightly varied, and the capacity of all the methods was found to remain unaffected by small deliberate variations. The results of this study are presented in Table 4 and indicate that the proposed methods are robust. Method ruggedness was demonstrated having the analysis done by four analysts and also by a single analyst performing analysis on four different apparatus or instruments in the same laboratory. Intermediate precision values (%RSD) in both instances were in the range of 0.59%–2.96% indicating acceptable ruggedness. The results are presented in Table 4. 

### 6.4. Selectivity

In the analysis of placebo blank, there was no measurable consumption of Ce(IV) in titrimetry and the same absorbance value as obtained for the reagent blank was recorded in method B and method C, suggesting the noninterference by the inactive ingredients added to prepare the placebo.

In method A, 5 mL of the resulting solution prepared by using synthetic mixture was assayed titrimetrically (*n* = 5) and yielded a percentage of recovery of 102.3 ± 0.62 KTF. In spectrophotometry, 2 mL of 20 *μ*g mL^−1^ KTF in method B and 3 mL of 20 *μ*g mL^−1^ KTF in method C when subjected to analysis (*n* = 5) yielded percentage of recoveries of 98.24% and 101.8% KTF with standard deviations of 1.74 and 2.32, respectively. These results complement the findings of the placebo blank analysis with respect to selectivity.

### 6.5. Application to Tablet Analysis

Commercial KTF tablets were analyzed using the developed methods and also a reference method [38]. The reference method involves potentiometric titration of KTF in anhydrous acetic anhydride—acetic acid medium with acetous perchloric acid. The results obtained were compared statistically by Student's *t*-test and the variance-ratio *F*-test. The calculated *t* and *F* values did not exceed the tabulated values of 2.78 and 6.39 at the 95 % confidence level and for four degrees of freedom, indicating close similarity between the proposed methods and the reference method with respect to accuracy and precision. These results are summarized in Table 5.

### 6.6. Recovery Study

To further ascertain the accuracy and reliability of the methods, recovery experiments were performed via standard-addition procedure. Preanalysed tablet powder was spiked with pure KTF at three different levels and the total was found by the proposed methods. Each determination was repeated three times. The percent recovery of pure KTF added (Table 6) was within the permissible limits indicating the absence of inactive ingredients in the assay.

## 7. Conclusions

Three methods have been developed for determination of ketotifen fumarate in bulk drug and in its tablets and validated as per the current ICH guidelines. The present visual titrimetric method is simple and economical compared to the reported coulometric method [23]. The spectrophotometric methods are characterized by simplicity since they do not involve any critical experimental variable and are free from tedious and time-consuming extraction steps and use of organic solvents unlike many previous methods as indicated in Table 1. The method using *o*-dianisidine looks more sensitive than most existing methods. Both have additional advantages of ease of operation and possibility of carrying them out with a common laboratory instrument unlike many other instrumental methods reported for ketotifen. They are characterized by high selectivity and comparable sensitivity with respect to the existing methods. The accuracy, reproducibility, simplicity, and cost-effectiveness of the methods suggest their application in the quality control laboratories where the modern and expensive instruments are not available. 

## Figures and Tables

**Table 1:** Comparison of the performance characteristics of the proposed methods with the existing visible spectrophotometric methods.

Serial number	Reagent(s) used	Methodology	*λ*_max⁡_ (nm)	Linear range (*µ*g/mL) (*ε* = L/mol/cm)	Remarks	Reference
1	(a) Molybdenum thiocyanate (b) DDQ	Methylene chloride extractable ion-pair complex measured	469.5 588	5–37.5 10–80	Employs unstable oxidant, narrow linear range	[24]

2	(a) F-C reagent (b) NBS-celestine blue (c) Sulphanilamide (d) Azocarmine G	Measurement of the absorbance of blue colored chromogen Unreacted NBS measured using celestine blue in acid medium Measurement of pink colored coupled product of drug with diazotized sulphanilamide with drug Chloroform extractable 1 : 1 ion-pair complex was measured	720 540 520 540	4–28 2–12 1–10 2.5–25	Multistep, unstable oxidant, strict pH control, and tedious extraction procedure	[25]

3	(a) Bromophenol blue (b) Bromothymol green (c) Bromothymol blue (d) Bromocresol purple	Chloroform extractable 1 : 1 ion-pair complex was measured	—	0	Required close pH control and narrow linear range and involved tedious time consuming extraction steps	[26]

4	Bromocresol green	Chloroform extractable 1 : 1 ion-pair complex was measured	423	5.15–61.91	Required close pH control and narrow linear range and involved tedious time consuming extraction steps	[27]

5	Ce(IV)-*p*-DMAB	Oxidation form of *p*-DMAB measured	460	0.4–8.0 (4.0 × 10^4^)	Simple, highly sensitive, extraction free, and use of stable cerium(IV) solution	This work
	Ce(IV)-*o*-dianisidine	Oxidation form of *o-*dianisidine measured	470	0.4–10 (3.7 × 10^4^)		

**Table 2:** Sensitivity and regression parameters.

Parameter	Method B	Method C
*λ*_max⁡_, nm	460	470
Colour stability, min	60	15
Linear range, *μ*g mL^−1^	0.4–8.0	0.4–10
Molar absorptivity (*ε*), L mol^−1^ cm^−1^	4.0 × 10^4^	3.7 × 10^4^
Sandell sensitivity*, *μ*g cm^−2^	0.0105	0.0136
Limit of detection (LOD), *μ*g mL^−1^	0.39	0.43
Limit of quantification (LOQ), *μ*g mL^−1^	1.19	1.32
Regression equation, Y**		
Intercept (*a*)	0.8744	0.8082
Slope (*b*)	−0.0914	−0.0815
Standard deviation of *a* (*S*_*a*_)	2.2 × 10^−2^	6.6 × 10^−2^
Standard deviation of *b* (*S*_*b*_)	4.9 × 10^−3^	7.3 × 10^−3^
Regression coefficient (*r*)	−0.9998	−0.9990

*Limit of determination as the weight in *μ*g per mL of solution, which corresponds to an absorbance of *A* = 0.001 measured in a cuvette of cross-sectional area 1 cm^2^ and *l* = 1 cm. **Y = *a* + *bX*, where *Y* is the absorbance, *X* is concentration in *μ*g mL^−1^, *a* is intercept, and *b* is slope.

**Table 3:** Results of intraday and interday accuracy and precision study.

Method	KTF taken mg/*µ*g mL^−1^	Intraday accuracy and precision (*n* = 7)			Interday accuracy and precision (*n* = 7)		
		KTF found mg/*µ*g mL^−1^	RE %	RSD %	KTF found mg/*µ*g mL^−1^	RE %	RSD %
A	5.00	5.06	1.20	1.80	5.12	2.42	2.90
	10.0	10.12	1.62	1.40	10.15	1.51	2.43
	15.0	14.80	1.34	0.94	14.72	1.58	1.92

B	2.0	2.02	1.23	1.72	2.03	1.62	1.18
	4.0	3.96	1.62	0.87	3.94	1.26	1.58
	6.0	6.08	1.33	1.46	6.17	1.90	0.88

C	4.0	3.94	0.73	1.43	3.95	1.14	2.12
	6.0	6.06	1.06	1.09	6.11	1.84	1.79
	8.0	7.90	1.21	1.18	7.88	1.47	1.09

In method A, KTF taken/found, is in mg and it is *µ*g mL^−1^ in method B and C.

RE: relative error (%); RSD: relative standard deviation (%).

**Table 4:** Method robustness and ruggedness expressed as intermediate precision.

Method	KTF taken mg/*µ*g mL^−1^	Robustness		Ruggedness	
		H_2_SO_4_ volume, mL (% RSD), *n* = 3	Reaction time, min (% RSD), *n* = 3	Ineranalysis (% RSD), *n* = 4	Intercuvettes/burettes (% RSD), *n* = 4
A	5.0	0.88	1.63	0.81	0.85
	10.0	1.33	1.83	1.93	1.23
	15.0	1.03	0.59	0.89	0.96

B	2.0	2.60	2.11	2.11	2.38
	4.0	2.71	1.36	2.63	1.82
	6.0	1.79	1.80	1.58	2.83

C	4.0	2.66	2.70	2.01	2.71
	6.0	2.45	1.83	2.79	1.99
	8.0	1.72	1.27	1.69	2.96

In titrimetry, standing times were 8, 10, and 12 min.

In spectrometric methods B and C, reaction time, 10 ± 1 min; volume of H_2_SO_4_ 2 ± 0.2 mL and 1 ± 0.2 mL varied, respectively.

**Table 5:** Results of analysis of tablets by the proposed methods and statistical comparison with the reference method.

Tablets analysed	Label claim, mg/tablet	Found* (percent of label claim ± SD)			
		Reference method	Proposed methods		
			Method A	Method B	Method C
Asthafen	1	100.8 ± 0.91	101.4 ± 0.89	102.8 ± 1.91	102.1 ± 1.75
			*t* = 1.05	*t* = 2.11	*t* = 2.16
			*F* = 1.05	*F* = 4.41	*F* = 3.7

Ketasma	1	102.3 ± 1.02	100.6 ± 0.98	102.8 ± 1.36	101.3 ± 0.88
			*t* = 2.69	*t* = 0.66	*t* = 1.66
			*F* = 1.08	*F* = 1.78	*F* = 1.34

*Mean value of five determinations;

Tabulated *t* value at the 95% confidence level is 2.78.

Tabulated *F* value at the 95% confidence level is 6.39.

**Table 6:** Results of recovery study via standard addition method with tablet.

Method	Tablet studied	KTF in tablet mg/*µ*g mL^−1^	Pure KTF added mg/*µ*g mL^−1^	Total found mg/*µ*g mL^−1^	Pure KTF* Percent ± SD
A	Ketasma-1	6.03	3.0	9.24	102.3 ± 1.98
		6.03	6.0	12.22	101.6 ± 1.25
		6.03	9.0	15.29	101.3 ± 2.08

B	Ketasma-1	3.03	1.5	4.54	101.1 ± 1.26
		3.03	3.0	6.09	102.1 ± 1.98
		3.03	4.5	7.59	101.4 ± 1.34

C	Ketasma-1	2.05	1.0	3.06	101. 1 ± 1.63
		2.05	2.0	4.03	99.36 ± 1.28
		2.05	3.0	5.12	102.4 ± 1.53

*mg in method A; *µ*g mL^−1^ in methods B and C. *Mean value of three determinations.

## References

[1] Sweetman SC (2005). *Martindale: The Complete Drug Reference*.

[2] Alali FQ, Tashtoush BM, Naji NM (2004). Determination of ketotifen in human plasma by LC-MS. *Journal of Pharmaceutical and Biomedical Analysis*.

[3] Dollery C (1999). *Therapeutic Drugs*.

[4] Abounassif MA, El-Obeid HA, Gadkariem EA (2005). Stability studies on some benzocycloheptane antihistaminic agents. *Journal of Pharmaceutical and Biomedical Analysis*.

[5] Chen GL, Qu H (2003). HPLC determination of ketotifen fumarate in compound metamizol sodium tablets. *Yaowu Fenxi Zazhi*.

[6] Nnane IP, Damani LA, Hutt AJ (1998). Development and validation of stability indicating high-performance liquid chromatographic assays for ketotifen in aqueous and silicon oil formulations. *Chromatographia*.

[7] Zarapkar SS, Bhounsule NJ, Halkar UP (1992). High performance liquid chromatographic determination of ketotifen I hydrogen fumarate in pharmaceuticals. *Indian Drugs*.

[8] Hoogewijs G, Massart DL (1984). Development of a standardized analysis strategy for basic drugs, using ion-pair extraction and high-performance liquid chromatography—III. Analysis of pharmaceutical dosage forms. *Journal of Pharmaceutical and Biomedical Analysis*.

[9] Muralidharan S, Han LB, Ming Y, Lau J, Sailishni K, Arumugam S (2012). Simple and accurate estimation of ketotifen fumarate by RP-HPLC. *International Journal of Pharmaceutical, Chemical and Biological Sciences*.

[10] Semreen MH (2005). Optimization and validation of HPLC method for the analysis of ketotifen fumarate in a pharmaceutical formulation. *Bulletin of Pharmaceutical Sciences, Assiut University*.

[11] Sanghavi NM, Puranik KA, Samarth MM (1995). Analysis of ketotifen fumarate by high performance thin layer chromatography. *Indian Drugs*.

[12] Kyung-Don N, Sung-Kwon T, Ji-Sun P (2012). Bioequivalence assessment of Fumatifen tablet to Zaditen tablet (ketotifen fumarate 1 mg) by liquid chromatography tandem mass spectrometry. *Journal of Pharmaceutical Investigation*.

[13] Zhou M, Li Y, Ma Y, Wang W, Mi J, Chen H (2011). Determination of ketotifen fumarate by capillary electrophoresis with tris(2,2′-bipyridyl) ruthenium(II) electrochemiluminescence detection. *Luminescence*.

[14] Sastry CSP, Naidu PY (1998). Spectrofluorimetric estimation of ketotifen and terfenadine in pharmaceutical formulations. *Indian Drugs*.

[15] Bersier PM, Szczepaniak W, Ren M (1992). Direct differential pulse polarographic and adsorptive stripping voltammetric assay of ketotifen in tablets. *Archiv der Pharmazie*.

[16] Mohamed ME, Aboul-Enein HY (1986). Spectrophotometric and differential pulse polarographic methods of analysis for ketotifen hydrogen fumarate. *Drug Development and Industrial Pharmacy*.

[17] Szczepaniak W, Cychowska T, Przadka T (1992). Spectrophotometric determination of ketotifen in pharmaceutical preparations after isolation on ion-exchanger.. *Acta poloniae pharmaceutica*.

[18] Hopkała H, Drozd J (1999). Ion-selective electrodes for the determination of antihistaminic drug ketotifen. *Chemia Analityczna*.

[19] Frag EYZ, Mohamed GG, Gehad, Khalil MM, Hwehy MA (2011). Potentiometric determination of ketotifen fumarate in pharmaceutical preparations and urine using carbon paste and PVC membrane selective electrodes. *International Journal of Analytical Chemistrypages*.

[20] Li LJ, Gao WY, Hu DC, Cai Z, Li YQ (2010). Electrochemiluminescence method for determination of ketotifen fumarate on glassy carbon electrode modified with platinum-multi-walled carbon nanotube modified electrode. *Fenxi Ceshi Xuebao*.

[21] Fei N, Jiuru L (2007). Determination of ketotifen by using calcein as chemiluminescence reagent. *Analytica Chimica Acta*.

[22] He SH, Tian KJ, Zhang SQ, Yu WY (2005). Determination of ketotifen fumarate based on the chemiluminescence reaction of luminol with ferricyanide. *Fenxi Ceshi Xuebao*.

[23] Ciesilski W, Zakrzewski R, Złobińska U (2005). Coulometric titration of ketotifen in tablets. *Pharmazie*.

[24] El-Kousy N, Bebawy LI (1999). Determination of some antihistaminic drugs by atomic absorption spectrometry and colorimetric methods. *Journal of Pharmaceutical and Biomedical Analysis*.

[25] Sastry CSP, Naidu PY (1997). Spectrophotometric estimation of ketotifen fumarate in pharmaceutical formulations. *Mikrochimica Acta*.

[26] Sane RT, Chonkar NL, Surve SR, Gangrade MG, Bapat VV (1993). Extractive colorimetrics estimation of (i) ticlopidine hydrochloride, (ii) buspirone hydrochloride, (iii) nefopam hydrochloride and (iv) ketotifen fumarate from pharmaceutical preparations. *Indian Drugs*.

[27] Amanlou M, Hoseinzadeh MH, Azizian H, Souri E, Farsam H (2007). Determination of Ketotifen fumarate in raw material and pharmaceutical products using ion-pair formation. *Analytical Letters*.

[28] Vachek J (1987). Photometric determination of Ketotifen. *Cesko-Slovenska Farmacie*.

[29] Devi OZ, Basavaiah K, Revanasiddappa HD, Vinay KB (2011). Titrimetric and spectrophotometric assay of pantoprazole in pharmaceuticals using cerium(IV) sulphate as oxidimetric agent. *Journal of Analytical Chemistry*.

[30] Rajendraprasad N, Basavaiah K, Tharpa K, Vinay KB (2009). Quantitative determination of olanzapine in tablets with visible spectrophotometry using cerium(IV)sulphate and based on redox and complexation Reactions. *Eurasian Journal of Analytical Chemistry*.

[31] Rajendraprasad N, Basavaiah K (2010). Highly sensitive spectrophotometric determination of olanzapine using cerium(IV) and iron(II) complexes of 1,10-phenanthroline and 2,2′-bipyridyl. *Journal of Analytical Chemistry*.

[32] Basavaiah K, Tharpa K, Rajendraprasad N, Hiriyanna SG, Vinay KB (2009). Sensitive cerimetric assay of olanzapine in pharmaceuticals. *Biomedical Journal*.

[33] Raghu MS, Basavaiah K (2012). Simple, sensitive and eco-friendly methods for the determination of methdilazine in tablets and syrup using cerium (IV). *Chemical Sciences Journal*.

[34] Vogel AI (1961). *A Text-Book of Quantitative Inorganic Analysis*.

[35] DasGupta BR, Boroff DA (1968). Quantitative spectrophotometric determination of hydrogen peroxide with para-dimethylaminobenzaldehyde. *Analytical Chemistry*.

[36] Christian GD (2007). *Text Book of Analytical Chemistry*.

[37] Validation of analytical procedures: text and methodology Q2 (R 1), complementary guideline on methodology.

[38] (2009). *British Pharmacopoeia: Volume I and II*.

